# Electroanalytical Paper-Based Nucleic Acid Amplification Biosensors with Integrated Thread Electrodes

**DOI:** 10.1021/acs.analchem.1c02900

**Published:** 2021-10-14

**Authors:** Shirin Khaliliazar, Anna Toldrà, Georgios Chondrogiannis, Mahiar Max Hamedi

**Affiliations:** School of Engineering Sciences in Chemistry, Biotechnology, and Health, KTH Royal Institute of Technology, Teknikringen 56, Stockholm 10044, Sweden

## Abstract

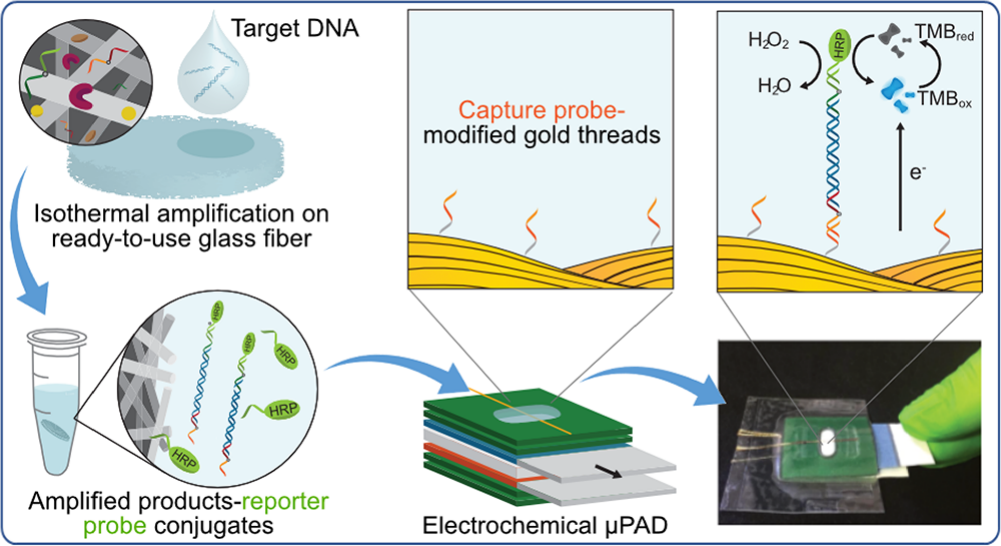

Nucleic acid amplification tests (NAATs) are very sensitive and specific methods, but they mainly rely on centralized laboratories and therefore are not suitable for point-of-care testing. Here, we present a 3D microfluidic paper-based electrochemical NAAT. These devices use off-the-shelf gold plasma-coated threads to integrate electroanalytical readouts using *ex situ* self-assembled monolayer formation on the threads prior to assembling into the paper device. They further include a sandwich hybridization assay with sample incubation, rinsing, and detection steps all integrated using movable stacks of filter papers to allow time-sequenced reactions. The devices use glass fiber substrates for storing recombinase polymerase amplification reagents and conducting the isothermal amplification. We used the paper-based device for the detection of the toxic microalgae *Ostreopsis* cf. *ovata*. The NAAT, completed in 95 min, attained a limit of detection of 0.06 pM target synthetic DNA and was able to detect 1 ng/μL *O.* cf. *ovata* genomic DNA with negligible cross-reactivity from a closely related microalgae species. We think that the integration of thread electrodes within paper-based devices paves the way for digital one-time use NAATs and numerous other advanced electroanalytical paper- or textile-based devices.

Nucleic acid amplification tests (NAATs) are highly sensitive and specific molecular techniques that identify particular sequences in a genetic material. Current NAAT diagnostics rely mainly on centralized polymerase chain reaction (PCR) tests performed by trained personnel in laboratories. The integration of NAATs into point-of-care (POC) devices can be ground-breaking and allow detection at the site and at scale, enabling rapid and appropriate decisions not only in public health but also in food safety and environmental monitoring.^1−5^

Microfluidic paper analytical devices (μPADs) have gained interest for NAAT POC devices because they have the potential to be disposable and portable. The use of various papers and other substrates provides properties necessary for the different steps of NAAT assays such as fluid transport through capillary forces, which eliminates the need for external pumps, filtration, adsorption, and reagent storage in the porous network of papers.^6−10^ μPADs also allow 3D microfluidic systems using multiple layers to integrate advanced functions.^11−14^

PCR, the gold standard DNA amplification method, requires thermocyclers to conduct a series of high-temperature cycles for several hours and is therefore not easy to integrate into POC paper-based devices. For this reason, isothermal amplification methods that operate at a constant temperature are considered more suitable options for paper-based NAATs.^8,15^ Among several isothermal amplification methods, recombinase polymerase amplification (RPA) emerges as an attractive method as it works at a low temperature of 37–42 °C and it completes amplification in a short time of 10–30 min.^16^ Besides these advantages, conducting RPA in porous matrices facilitates further integration with μPADs.^17,18^

To read out the results of isothermal reactions in paper-based devices, previous works have mainly relied on colorimetric methods,^19−23^ which suffer from low sensitivity, are semiquantitative, and require human operators or optical instruments.^24^ To enable quantification, miniaturization, and integration, few paper-based NAATs have been combined with electronic readout using redox molecules in solution that bind to dsDNA.^25,26^ These devices however are not sequence-specific, compromising their sensitivity. Electrochemical DNA hybridization biosensors can overcome these issues. They have a high sensitivity and specificity because they are based on hybridization events to generate signals in close contact with a DNA-modified transducer.^27,28^ A number of DNA-immobilized biosensors have to date been presented for isothermal techniques in general and RPA specifically, exploiting different detection configurations like labeled capture probes,^29,30^ labeled amplified products,^31^ or labeled reporter probes,^32,33^ as well as solid-phase strategies.^34−37^ Despite the outstanding advantages of these electroanalytical biosensors, no such device has so far been integrated in μPADs for isothermal NAATs.

The reason for this is that paper-based electroanalytical DNA devices so far rely on printing technology to mostly print carbon electrodes.^38−43^ To subsequently *in situ* functionalize these electrodes with DNA probes, carbon electrodes are modified either chemically,^41,43^ with carbon nanomaterials^44,45^ and/or metals^46,47^ (including gold^26,38,48,49^), making the process cost- and time-inefficient for large-scale fabrication. They are further irreproducible and prone to contamination, which has hindered their use for DNA probe integration for NAATs.

Contrary to printed carbon, gold is the most desirable electrode material because of its inert properties and the simple, one-step formation of self-assembled monolayers (SAMs) via thiol-modified DNA probes.^50^ As demonstrated by Crooks’ group,^51^ gold-coated microfibers can be integrated within μPADs, offering two main advantages: (i) they are easier to incorporate than typical screen-printed carbon electrodes; (ii) they can be *ex situ* cleaned and functionalized before device fabrication. Off-the-shelf gold plasma-coated threads are highly flexible and conductive fiber electrodes that do not require any cleaning step before SAM modification, making them suitable for mass-produced electrodes as we recently reported.^29,52^

Here, we report a multilayer paper-based electrochemical DNA sensor with integrated electrodes in the form of gold threads. This 3D μPAD shown in Figure 1 includes different layers (hollow channels, movable wax barriers, and movable absorbent pad) allowing time-sequence retentions, flow, and absorption of solutions to conduct a sandwich hybridization assay (SHA) with minimum user intervention. We combined RPA on glass fiber substrates with the integrated electrochemical μPAD to specifically detect the toxic microalgae *Ostreopsis* cf. *ovata*, which harms marine life and human health.^53^ Our test offers advantages in terms of design, operation, and sensitivity compared with existing electrochemical μPADs and shows strong potential for digital single-use POC testing.

**Figure 1:**
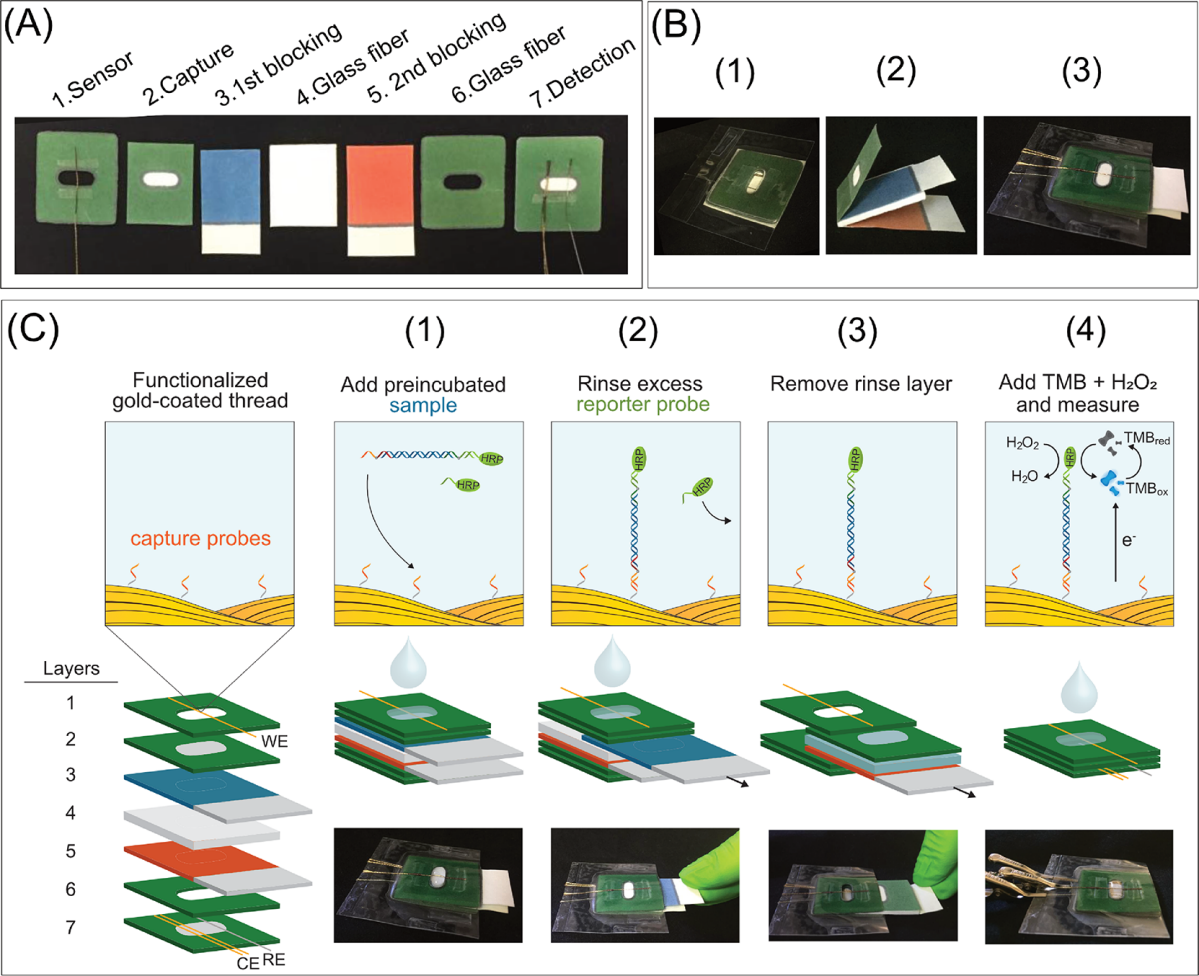
Photos and schematics of the components, fabrication, and operation of the device. (A) Components and functions: (1) Sensor: a wax layer with a hollow channel in which a capture probe-modified gold thread working electrode (WE) is placed; (2) Capture: a wax layer with the hydrophilic paper channel; (3) 1st blocking: a movable wax layer with a flange; (4) Glass fiber: a layer of glass fiber absorbent pad; (5) 2nd blocking: a movable wax layer with a flange; (6) spacer: a wax layer with a hollow channel; and (7) Detection: a wax layer with a hydrophilic paper channel in which the gold thread counter electrode (CE) and silver thread pseudo-reference electrode (RE) are located. (B) Fabrication: (1) Detection, spacer, and sensor layers are aligned and laminated; (2) The movable layers are prepared: the capture, glass fiber, and 2nd blocking layers are taped together, and the 1st blocking layer is then placed between the capture and glass fiber layers; and (3) The movable layers are inserted inside the laminated layers (between the detection and spacer layers), and the functionalized working electrode is placed on the detection layer. (C) Operation: (1) The sample of RPA product-HRP conjugates is added to the top of the device and hybridized with the capture probe on the gold thread; (2) The 1st blocking layer is manually removed; (3) Following a washing step with Milli-Q water, the capture/glass fiber/2nd blocking layers are simultaneously removed, bringing the sample to the three-electrode sensor layer; and (4) The TMB + H_2_O_2_ enzymatic substrate is added, and chronoamperometric measurements are carried out.

## Experimental Section

### Chemicals and Materials

We purchased phosphate-buffered saline (PBS) tablets (10 mM phosphate saline, 2.7 mM potassium chloride, 137 mM sodium chloride, pH 7.4), potassium ferricyanide K_3_[(FeCN)_6_], and skim milk powder from Sigma Aldrich (Sweden). We purchased nuclease-free water, 10× Tris-borate-EDTA (TBE) buffer, and Pierce TMB enzymatic substrate for horseradish peroxidase (HRP) containing TMB (3,3′,5,5′-tetramethylbenzidine) and H_2_O_2_ from Thermo Fisher Scientific (Sweden). We used a TwistAmp Basic RPA kit from TwistDX Limited (UK) to conduct isothermal amplification and a QIAquick PCR Purification Kit from QIAGEN (Germany) to purify amplified products. Custom DNA oligonucleotides were synthesized by Biomers (Germany). We purchased Whatman grade 1 filter paper from Fisher Scientific (Sweden), and Whatman CF7 glass fiber pads were provided by Cytiva (Sweden). We purchased gold and silver multifilament threads from Swicofil (Switzerland) (see Table S1 for the specifications of the electrode threads).

### Oligonucleotide Sequences and Microalgal Genomic DNA

We used primers and probes previously reported by Toldrà et al. (Table 1).^32^ Primers for *O.* cf. *ovata* (designed within the ribosomal DNA genes) were modified with tails, and probes were complementary to these tails: a thiolated capture probe and an HRP-labeled reporter probe.

**Table 1:** List of Oligonucleotide Sequences and Their Respective Modifications (Underlined)

name	sequence (5′-3′)
*O.* cf. *ovata* FwP with tail	gtt ttc cca gtc acg ac-C3-aca atg ctc atg cca atg atg ctt gg
*Ostreopsis* spp. RvP with tail	tgt aaa acg acg gcc agt-C3-gca wtt ggc tgc act ctt cat aty gt
thiolated capture probe	gtc gtg act ggg aaa act ttt ttt ttt ttt tt-C3-SH
HRP-labeled reporter probe	HRP-act ggc cgt cgt ttt aca
*O.* cf. *ovata* target	aca atg ctc atg cca atg atg ctt ggt ggc atg cac ctt gtt agt tgt agc atg aca gct tga tac tta tct aaa cgc ttt cat caa ctg tct tct gac agc aat gaa tgc atc aat tca aaa caa tat gaa gag tgc agc caa atg c

We used extracted and purified genomic DNA from strains *O.* cf. *ovata* IRTA-SMM-16-133 (GenBank reference: MH790463) and *Ostreopsis* cf. *siamensis* IRTA-SMM-16-84 (GenBank reference: MH790464) as target and nontarget controls, respectively, which were gently provided by IRTA (Spain).

### Recombinase Polymerase Amplification

We performed RPA in solution and in porous matrices. In both cases, we conducted RPA in triplicate at 37 °C for 30 min following concentrations previously optimized and reported:^32^ 14.75 μL of rehydration buffer, 22.95 μL of nuclease-free water, 1/2 enzyme pellet, 2.4 μL of each primer (10 μM), 2.5 μL of magnesium acetate (480 mM), and 5 μL of DNA or nuclease-free water.

We first performed RPA in 0.2 mL tubes using a heat block for positive (10 pM target synthetic DNA) and blank (nuclease-free water) samples. Then, we purified the RPA products using the QIAquick PCR Purification Kit following manufacturer instructions,^54^ with a final elution with 50 μL of the elution buffer of the kit.

In the next step, we carried out RPA in glass fiber substrates (0.6 × 0.4 cm with polyimide adhesive tape underneath) with the freeze-dried RPA master mix. We first freeze-dried the glass fiber substrates that contained the RPA master mix (including all the reagents except DNA or nuclease-free water and magnesium acetate) using a freeze drier (LaboGene, Denmark). After lyophilization, we conducted the RPA reaction by rehydrating the freeze-dried RPA master mix with a solution containing 42.5 μL of nuclease-free water, 5 μL of target DNA or nuclease-free water, and 2.5 μL of magnesium acetate. Reactions were performed in an INCU-Line incubator (VWR, Sweden) and included the following: (1) positive samples: 10-fold serial dilutions of target synthetic DNA (from 10 to 0.001 pM) or 1 ng/μL *O.* cf. *ovata* genomic DNA; (2) nontarget samples: 1 ng/μL *O.* cf. *siamensis* genomic DNA; or (3) blanks: nuclease-free water.

Additionally, we stored the freeze-dried master mix in glass fiber substrates for 1 week at −20 °C and then carried out the RPA reaction using 10 pM target synthetic DNA to examine the stability of the freeze-dried RPA master mix in glass fiber substrates. We also performed RPA in wax-printed Whatman Grade 1 filter papers (with reaction zones of 5 mm diameter) for 10 pM target synthetic DNA and blank samples following the aforementioned protocol for the RPA reaction in glass fiber substrates. We confirmed the efficiency of the RPA reaction through gel electrophoresis using 3% agarose in TBE (0.5X) buffer and stored all the RPA products at −20 °C until use.

### Preparation of Thread Electrodes and SAM Functionalization

We used gold and silver threads as received, without any cleaning procedure, and defined a length of 5 mm using nail polish for both threads. We incubated the gold working threads in 250 μL of 500 nM thiolated capture probe in PBS (1×) overnight (for at least 16 h) at 4 °C. After incubation, we rinsed the modified thread electrodes with Milli-Q water and kept them in PBS (1×) solution until analysis.

### Fabrication of a 3D μPAD

We created the device designs using AutoCAD (Autodesk Inc., USA). We first wax-printed the Whatman Grade 1 filter paper using a wax printer (Xerox colorQube 8570/8870, Malaysia) and then melted the wax patterns using an oven (VWR Ventil-Line, Germany) at 110 °C for 5 min. Then, we cut the patterns using a cutting machine (Brother ScanNCut CM900, China). Wax-printed filter papers included wax layers with oval (7 × 14 mm) hollow channels, wax layers with oval (7 × 14 mm) hydrophilic channels, and plain hydrophobic wax layers.

The device consisted of seven layers (Figure 1A): (1) The sensor layer (24 × 27 mm): a wax layer with a hollow channel; (2) The capture layer (20 × 25 mm): a wax layer with a hydrophilic paper channel; (3) The 1st blocking layer (18 × 33 mm): a movable wax layer with a flange; (4) The glass fiber layer (20 × 25 mm): a layer of glass fiber absorbent pad; (5) The 2nd blocking layer (20 × 35 mm): a movable wax layer with a flange; (6) The spacer layer (24 × 27 mm): a wax layer with a hollow channel; and (7) The detection layer (24 × 27 mm): a wax layer with a hydrophilic paper channel where a gold thread counter electrode and silver thread pseudo-reference electrode were placed.

We fabricated the device in three steps (Figure 1B). First, we aligned the detection, spacer, and sensor layers with double-sided tape and then laminated them between two plastic substrates using a hot press (Skilte Produktion E-15S, China). Second, we taped the capture/glass fiber/2nd blocking layers together as one movable layer. We inserted the movable 1st blocking layer between the capture and glass fiber layers. Third, we placed the SAM-modified gold thread as a working electrode (see Preparation of Thread Electrodes and SAM Functionalization) onto the top laminated layer of the device and finally inserted the taped movable layer with the 1st blocking layer inside the laminated layers.

### Sandwich Hybridization Assay

We conducted the SHA first in tubes and then in the paper-based electrochemical device. For the tube-based assay, electrochemical detection was performed in a bulk solution (tube-based detection system) and paper (paper-based detection system).

For the tube-based assay, we pre-incubated 50 μL of the purified RPA product (obtained from RPA in solution) with 50 μL of the 20 nM HRP-labeled reporter probe prepared in 2% w/v skim milk in PBS (1×) for 30 min under shaking at room temperature. Subsequently, we incubated the mixture with the functionalized gold threads using 0.2 mL tubes for 30 min under shaking at room temperature. Finally, we rinsed the incubated gold threads with Milli-Q water for 10 s to remove excess of the reporter probe and let them shortly air-dried. Chronoamperometric measurements (see Electrochemical Measurements) were performed in two detection systems: (1) Tube-based detection system: we added 1.5 mL of TMB + H_2_O_2_ enzymatic substrate to an Eppendorf tube containing the working, counter, and pseudo-reference thread electrodes, waited for 5 min, and then carried out the electrochemical measurements; and (2) Paper-based detection system: we added 80 μL of the enzymatic substrate to the device (composed of the detection, spacer, and sensor layers), waited for 5 min, and then carried out the electrochemical measurements.

For the integrated SHA in the paper-based device using freeze-dried RPA on the glass fiber substrates, we placed the glass fiber substrate containing RPA products into a 1.5 mL Eppendorf tube and directly added 50 μL of PBS (1×) and 50 μL of the 20 nM HRP-labeled reporter probe in 2% w/v skim milk in PBS (1×) (volume enough to cover the glass fiber substrate). We pre-incubated the mixture at room temperature for 30 min under shaking to allow diffusion of the RPA products and subsequent hybridization with the HRP-labeled reporter probes without any purification step in between. Then, we added 100 μL of this mixture into the inlet of the device, where the capture probe-modified gold thread was embedded, to allow hybridization between capture probes and RPA product-HRP conjugates. After 30 min at room temperature, we removed the 1st blocking layer to let the sample wick from the sensor layer to the glass fiber layer. Next, we rinsed the thread working electrode on the device by simply adding 100 μL of Milli-Q water to the inlet and waited for a few minutes to be completely absorbed by the rinse layer. We then removed the taped movable layer (capture/glass fiber/2nd blocking layers) to bring the working electrode on the top layer (the sensor layer) in contact with the integrated two gold threads as counter electrodes and one silver thread as a pseudo-reference electrode on the bottom layer (the detection layer). Finally, we added 80 μL of the TMB + H_2_O_2_ enzymatic substrate, waited for 5 min, and then carried out the chronoamperometric measurements (see Electrochemical Measurements).

### Electrochemical Measurements

We conducted electrochemical measurements using an Autolab PGSTAT204N with the MUX 16 module (Metrohm Autolab, Sweden) and the accompanying NOVA 1.11 software package.

We calculated the electrochemical effective surface area of the gold threads (for a 5 mm geometric area) and investigated the electrochemical performance of the unmodified and capture probe-modified thread electrodes in the tube- and paper-based systems by conducting cyclic voltammetry (CV) in 5 mM potassium ferricyanide solution at scan rates from 10 to 200 mVs^–1^.

We used volumes of 1.5 and 80 μL of the TMB + H_2_O_2_ enzymatic substrate to conduct the chronoamperometric measurements for the tube- and paper-based configurations, respectively. For both assays, after adding the enzymatic substrate, we waited for 5 min and then carried out the chronoamperometric measurements by applying a reducing potential of −0.1 V for 1 s and reading the current output. CVs in TMB/H_2_O_2_ showed that a potential lower than 0.15 V induced the complete reduction of TMB. The working potential of 0.1 V was chosen for chronoamperometry as we obtained the best discrimination between blank and positive samples.

### Data Analysis

We presented the experimental data as mean ± standard deviation (SD) for at least three independent samples (*n* ≥ 3) using Origin 9.1 (OriginLab Corporation, USA). Statistical analysis between two groups was conducted using Student’s *t*-test through Microsoft Excel 2016, considering statistically significant a *p*-value ≤ 0.05. We determined the LOD from the calibration curve fitted to the sigmoidal logistic four-parameter equation as the concentration corresponding to the nontarget controls plus three times their SD.

## Results and Discussion

### Strategy and Device Design and Operation

Our assay configuration exploited tailed primers for the RPA. A tailed primer consists of a single-stranded DNA (ssDNA) sequence (“tail”) that is added to the primer using a 3-C alkyl chain spacer, which prevents elongation of the tail during amplification. This results in duplex products flanked by ssDNA tails, which can be detected in a so-called SHA using capture and reporter probes complementary to the tails. Tailed primers and SHA have been reported for RPA,^32^ and we chose this strategy for our device because of the following: (i) It avoids post-amplification steps (e.g., digestion) prior to detection to generate single-stranded sequences able to hybridize to the surface-immobilized probe; and (ii) It is highly specific as it involves two hybridization events through capture and reporter probes.

We designed a 3D μPAD composed of seven different layers, each contributing one specific function, to integrate the SHA assay and electrochemical detection in the μPAD as illustrated in Figure 1A. The function of each layer in this device is as follows: (1) The sensor layer is a wax-printed filter paper with a hollow channel, containing the capture probe-modified gold thread working electrode and acting as the inlet; (2) The capture layer is a wax-printed filter paper with a hydrophilic paper channel facilitating vertical quick capillary flow of the sample fluid to the glass fiber absorbent pad; (3) The 1st blocking layer is a movable wax-barrier layer that stops/activates the fluid flow to enable timely incubation of the sample; (4) The glass fiber layer is a glass fiber pad that absorbs the sample fluid and facilitates a washing step; (5) The 2nd blocking layer is a movable wax-barrier layer that stops the flow, preventing the sample fluid to flow to the following layers; (6) The spacer layer is a wax-printed filter paper with a hollow channel, which provides depth to the detection zone and prevents direct contact or contamination of thread electrodes on the subsequent layer; and (7) The electroanalytical detection layer is a wax-printed filter paper with a hydrophilic paper channel that incorporates two gold threads and a silver thread as counter and pseudo-reference electrodes, respectively.

The device operation briefly involves four simple steps as depicted in Figure 1C: (1) The pre-incubated sample of the RPA product with the hybridized HRP-labeled reporter probe is added to the inlet on the top layer. In this step, the RPA product-HRP conjugates bind to the immobilized capture probe on the working electrode through their ssDNA tail; (2) The 1st blocking layer is removed, and the sample fluid flows vertically to the absorbent pad. To rinse the HRP-labeled reporter probe in excess, Milli-Q water is added to the inlet, and after a few minutes, it is completely absorbed by the pad; (3) The 2nd blocking layer together with the glass fiber and capture layers are removed, bringing the working electrode in contact with the embedded gold thread and silver thread electrodes; and (4) The TMB + H_2_O_2_ enzymatic substrate is added, and after 5 min, chronoamperometric measurements are carried out.

### Recombinase Polymerase Amplification

**Figure 2:**
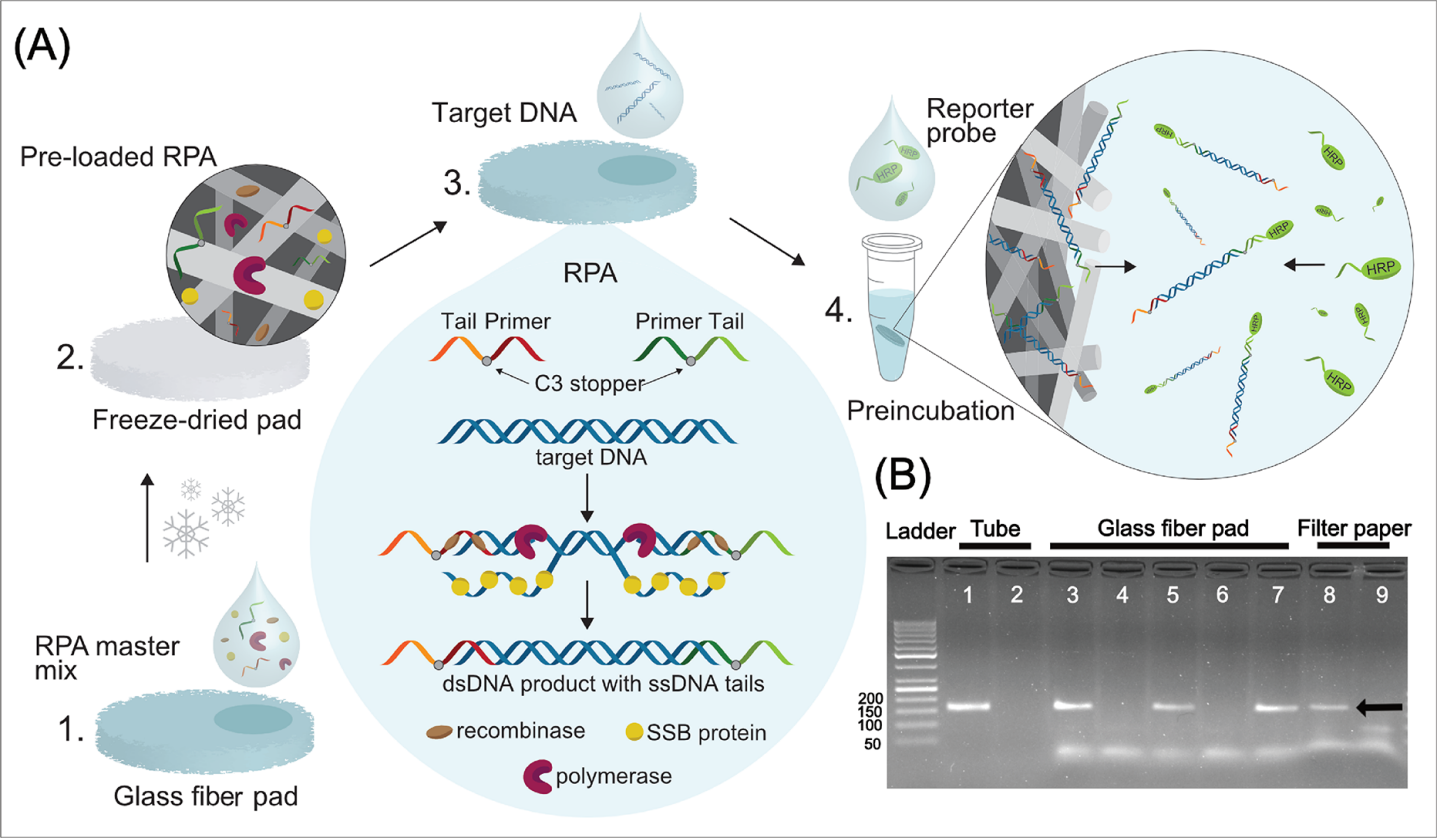
Schematic of the procedure for the RPA in glass fiber and gel electrophoresis of RPA products. (A) Procedure for the RPA in glass fiber: (1) RPA master mix is added in the glass fiber substrate; (2) The substrate is freeze-dried; (3) The target DNA is added, and RPA is performed at 37 °C for 30 min. The use of tailed primers results in dsDNA RPA products with ssDNA tails at each end; and (4) The substrate is pre-incubated for 30 min with a solution of the HRP-reporter probe. The RPA products diffuse from the paper to the solution and hybridize to the HRP-reporter probe through complementary tails, generating RPA product-HRP conjugates. (B) Agarose gel electrophoresis image of RPA products (148 bp dsDNA +35 bp ssDNA): 1 (positive with 10 pM target synthetic DNA, in solution and purified); 2 (blank, in solution and purified); 3 (positive with 10 pM target synthetic DNA, in glass fiber); 4 (blank, in glass fiber); 5 (positive with 1 ng/μL *O.* cf. *ovata* genomic DNA, in glass fiber); 6 (nontarget control (NC) with 1 ng/μL *O.* cf. *siamensis* DNA, in glass fiber); 7 (positive with 10 pM target synthetic DNA, in glass fiber after 1 week at −20 °C); 8 (positive with 10 pM target synthetic DNA, in Whatman filter paper); and 9 (blank, in Whatman filter paper).

To verify the performance of RPA with the tailed-modified primers, we conducted the RPA reaction in solution for 10 pM synthetic target DNA or blank samples and subsequently purified the RPA products using a conventional spin column purification kit. Gel electrophoresis results confirmed a successful RPA for the tailed-modified primers with the specific target band (148 bp dsDNA +35 bp ssDNA) (Figure 2B).

To further perform the RPA reaction in porous matrices, we evaluated two different materials: glass fiber and Whatman filter paper. We first freeze-dried the RPA master mix in these substrates and conducted the RPA for positive (10 pM target synthetic DNA) and blank samples at 37 °C in filter paper (5 mm diameter) and glass fiber (0.6 × 0.4 cm). We calculated the dimensions of the glass fiber substrates by measuring the area needed to absorb 50 μL volume of RPA. As seen in Figure 2B, the glass fiber substrates provided a stronger target band without nonspecific amplification products (e.g., primer-dimers) when compared to the paper substrates. These findings are consistent with Rohrman and Richards-Kortum’s study, which indicated the better yield and performance of the RPA reaction in glass fiber substrates than cellulose paper.^18^

Gel electrophoresis also showed specific bands for target *O. cf. ovata* genomic DNA and displayed no sign of amplification for nontarget *O. cf. siamensis* genomic DNA. We also demonstrated that the freeze-dried master mix in glass fiber was stable for 1 week at −20 °C, seen by the bright and specific band in Figure 2B (well 7) using 10 pM target synthetic DNA. These results demonstrate the utility of glass fiber as a ready-to-use substrate containing all the reagents necessary for RPA at the desired concentration, with the potential to be fully integrated within paper devices.

### Threads as Electrodes for DNA Sensing: Tube- and Paper-Based Detection Systems

**Figure 3:**
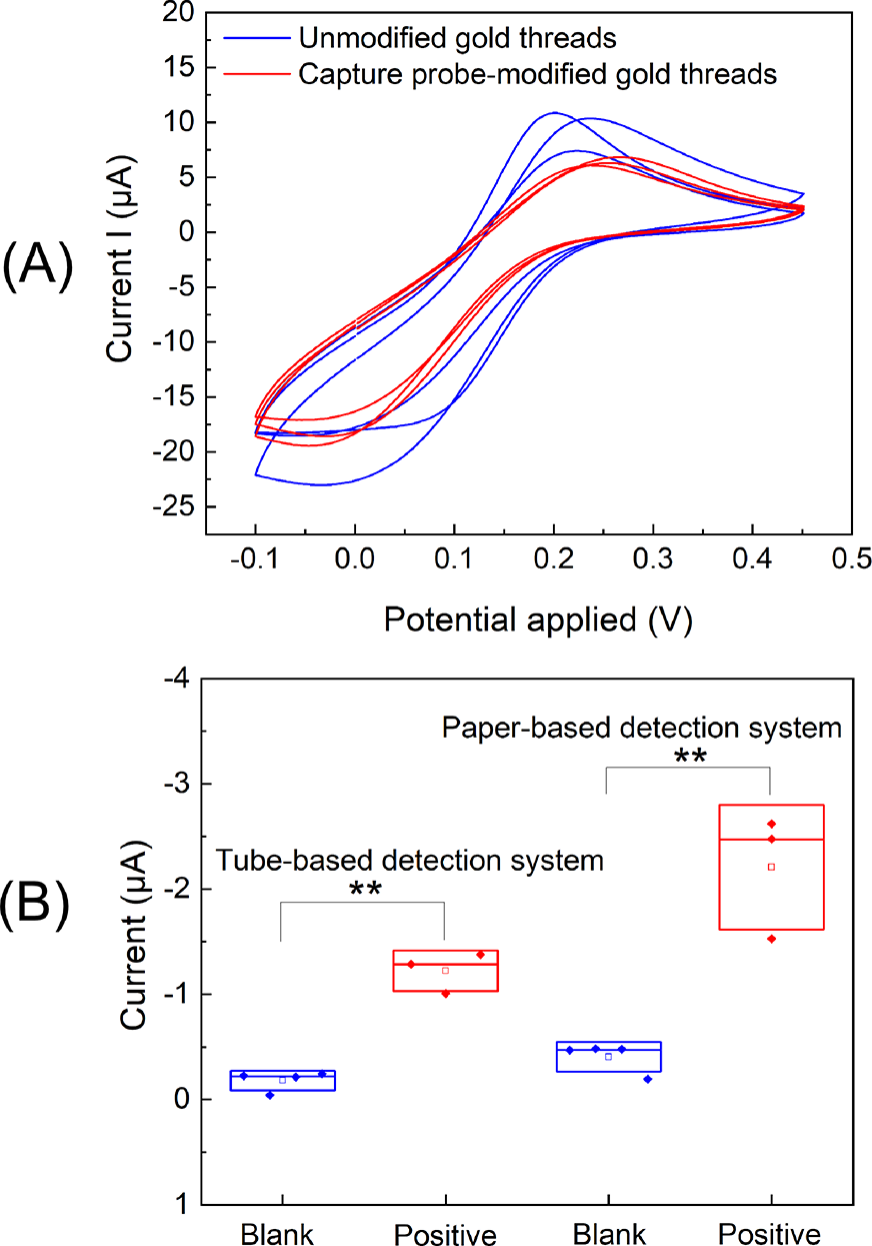
Characterization of gold threads and results of the thread-based DNA sensors. (A) Cyclic voltammograms of unmodified and capture probe-modified gold threads (*n* = 3) in potassium ferricyanide solution in the tube-based detection system at 50 mV s^–1^. (B) Chronoamperometric results of tube-based (*n* ≥ 3) and paper-based (*n* ≥ 3) detection systems for positive (10 pM target synthetic DNA) and blank (nuclease-free water) samples after the RPA reaction in solution and SHA in the tube.

Previously, we have reported the formation of SAMs on gold plasma-coated polyester multifilament threads and their use as electrodes.^29,52^ The formation of SAMs on these threads required no prior cleaning steps of the gold thread electrodes, making these materials ideal for SAM-based sensors to be fabricated at scale. In this study, we used the threads in combination with paper to endow μPADs, which to our knowledge has not been done before. We performed CVs of unmodified and capture probe-modified gold threads in potassium ferricyanide to characterize the coverage of SAMs, as they inhibit the electron transfer at the interface between the redox molecules in the solution and the electrode surface, resulting in lower redox peaks in the CVs (Figure 3A and Figures S1 and S2). The threads provided a 1.1 mm^2^ electrochemical effective surface area, which is reduced to 0.3 mm^2^ after its functionalization, corresponding to 74% probe coverage. We calculated these electrochemical effective surface areas using the Randles–Sevcik plots.

Next, we evaluated the feasibility of the threads to support the SHA in a tube-based assay using purified RPA products. We first pre-incubated the purified RPA products including positive (10 pM target synthetic DNA) and blank samples with the HRP-labeled reporter probe for 30 min to allow the tailed RPA products to hybridize to the complementary HRP-labeled reporter probes. We then added the capture probe-labeled gold thread electrodes to the pre-incubated RPA samples and incubated them for 30 min to allow the RPA product-HRP conjugates to hybridize to the immobilized capture probes on the electrode surface through their complementary tails. We rinsed the electrodes with Milli-Q water to remove unbound reporter probes and conducted chronoamperometric measurements using two different detection systems: tube- and paper-based systems. Using a three-electrode setup in an Eppendorf tube containing 1.5 mL of the TMB + H_2_O_2_ enzymatic substrate, chronoamperometric results showed a statistically significant difference between the positive and blank samples after 5 min of incubation (Figure 3B). Once the thread-based SHA assay in the tube-based detection system could successfully detect the purified RPA products, we followed the same protocol but performed the detection step in the paper-based system where electrodes were embedded. In this case, we performed chronoamperometric measurements after the addition of 80 μL of the enzymatic substrate to the inlet. The chronoamperometric results for the paper-based detection system in Figure 3B also showed a significant statistical difference between positive and blank samples.

Surprisingly, the chronoamperometric responses of the sensor with paper-based detection were higher than those obtained in the tube-based system. To study this difference, we performed two additional experiments. First, we conducted chronoamperometry in paper using a higher volume of the enzymatic substrate. As also highlighted by other authors,^55^ the use of a lower volume of the enzymatic substrate gave higher current intensities (Figure S3). Second, we compared Randles–Sevcik plots for the capture probe-modified gold threads in the paper and tube devices in the presence of potassium ferricyanide. The electrochemical reaction in the paper-based system at the surface of the capture probe-modified gold threads can be characterized by a faster electron transfer than in the tube system^56^ (Figure S4). Moreover, we studied the electrochemical behavior of unmodified gold threads in the tube- and paper-based systems (Figure S5). The use of different volumes of TMB + H_2_O_2_ for the tube- and paper-based detection systems may explain the higher current intensities observed for the latter, although the effect of fast electron transfer for SAM-modified threads in the paper cannot be ruled out.

### Integrated Paper-Based Electrochemical DNA Sensor

We proceed with the integration of the SHA in the electrochemical μPAD. We also carried out the RPA in glass fiber substrates to simplify steps before detection showing its potential to be integrated into sample-to-answer paper-based electrochemical NAATs.

We performed RPA reactions in the glass fiber substrates for positive (from 10 to 0.001 pM target synthetic DNA and 1 ng/μL*O.* cf. *ovata* genomic DNA), nontarget (1 ng/μL *O.* cf. *siamensis* genomic DNA), and blank samples. After amplification inside the glass fiber substrates, we directly pre-incubated the substrates containing the RPA products in a solution of the HRP-labeled reporter probe, without any intermediate purification step, which eliminated the need for several centrifugation steps. The diffused RPA products from the substrates hybridized with the reporter probes in the solution in a single step (Figure 2A). After this multipurpose step, we loaded the pre-incubated mixture containing RPA product-HRP conjugates with the reporter probe in excess into the inlet of the device where sample hybridization with immobilized capture probes occurred. The assay proceeded by adding the sample to the removable layer, *in situ* rinsing step, and addition of the enzymatic substrate to conduct the chronoamperometric measurements, as illustrated in Figure 1C.

**Figure 4:**
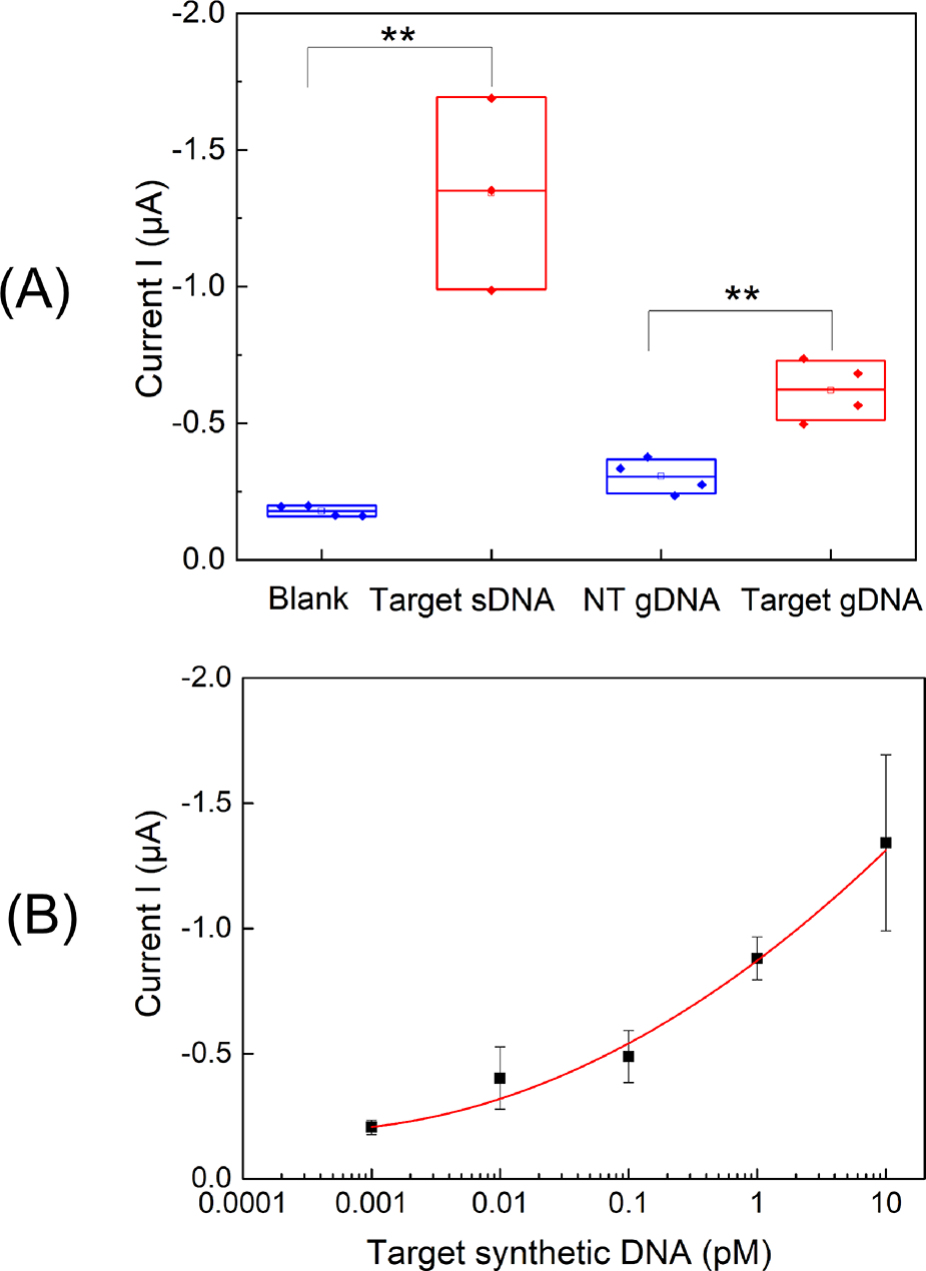
Chronoamperometric results of the SHA-integrated paper-based electrochemical sensor in the presence of unpurified RPA products in freeze-dried glass fiber substrates. (A) Chronoamperometric results (*n* ≥ 3) for blank (nuclease-free water), target sDNA (10 pM target synthetic DNA), NT gDNA (1 ng/μL nontarget *O.* cf. *siamensis* genomic DNA), and target gDNA (1 ng/μL target *O.* cf. *ovata* genomic DNA). (B) Calibration curve based on the sensor responses for the RPA products obtained from 10 to 0.001 pM target synthetic DNA (*n* = 3).

The obtained results indicate that the paper-based sensor specifically detected the unpurified RPA target products, showing a significant statistical difference between RPA products of target synthetic DNA (10 pM) and blank samples (Figure 4A). We also analyzed more complex samples containing genomic DNA that had been extracted from microalgal cultures. Positive samples containing target *O.* cf. *ovata* genomic DNA showed a significant statistical difference compared to samples containing nontarget *O.* cf. *siamensis* genomic DNA (Figure 4A). These results demonstrate the specificity of the biosensor, able to detect the target region in a complex matrix with no interferences from a close related microalgae species (i.e., *O.* cf. *siamensis*) from the same genus. In fact, since *O.* cf. *ovata* and *O.* cf. *siamensis* share similar gene sequences (even one of the primers is specific for both species, see Table 1), this suggests that the sensor will also be specific toward other nonclosely related species. The slightly higher signal observed in the nontarget samples compared to the blank samples (Figure 4A) has also been reported in previous studies.^32^ Since the nontarget control is more realistic, we used this value to determine the LOD of our test from the calibration curve with target synthetic DNA added to the freeze-dried RPA reaction in glass fiber substrates (Figure 4B), obtaining a concentration of 0.06 pM. Considering that one cell of *O.* cf. *ovata* has 2137 ribosomal DNA copies per cell,^57^ our assay is able to detect as low as 85 microalgal cells per sample, without taking into account sample collection and DNA extraction steps. Therefore, the biosensor may enable quantifications of *O.* cf. *ovata* below the current alarm thresholds proposed for *Ostreopsis* cells (10,000–30,000 cells/L seawater).^58^

The total operational time from sample to answer for the paper-based electrochemical sensor was 95 min and required four steps in total: (1) RPA (30 min); (2) Pre-incubation (30 min); (3) Incubation of the RPA product-HRP conjugates with the capture probe (30 min); and (4) Electrochemical detection (5 min). The 3D architecture of the paper device allowed us to simplify operation and bypass fluidic manipulation by the user for the washing steps of SHAs. It however still requires user intervention to transfer the enzymatic substrate to the paper device and two manual steps of valving with the blocking layers.

## Conclusions

We have described an electrochemical paper-based NAAT with integrated threads as electrodes. Our device has three advantages over other reported electrochemical nucleic acid biosensor μPADs: (i) It allows straightforward and *ex situ* SAM formation on gold thread electrodes prior device fabrication, which has not been reported before for μPADs. Subsequent integration of the SAM-coated electrodes within a paper design results in a better performance in terms of both current response and electron transfer than electrodes in solution; (ii) It requires minimum user intervention by using movable paper layers and uses the fiber substrates to store the necessary reagents for DNA amplification; and (iii) Its assay configuration exploits the use of tailed primers to avoid any post-amplification treatment, which is otherwise necessary for producing ssDNA, as required in all electrochemical sensors based on conformational switches.^38^ Moreover, our sandwich-type approach involves two hybridization events, which helps increase the specificity of our assay compared to other electrochemical μPADs.^38,41−43^ Combined with isothermal DNA amplification, this assay results in both high specificity and high sensitivity: we attain an LOD of 0.06 pM target synthetic DNA and is able to specifically detect 1 ng/μL *O. cf. ovata* genomic DNA with negligible cross-reactivity from a closely related microalgae species. The reported electrochemical DNA hybridization μPADs do not include an amplification step,^38−43^ resulting in higher LODs (commonly in the nanomolar–picomolar range), which are too high for most early detection of diseases.

To the best of the authors’ knowledge, this is the first report describing off-the-shelf gold threads for DNA sensing. We think that the integration of the off-the-shelf threads within paper-based devices paves the way for sensitive and specific digital POC NAATs and numerous other advances for electroanalytical devices in μPADs, even beyond DNA sensing. The turn-around time for completing the presented μPAD is close to that of the time for instrument-based PCR assays. Nevertheless, our isothermal amplification and electroanalytical detection embedded in paper microfluidics require less manual intervention and, more importantly, has the potential to be portable, facilitating testing in resource-limited areas, which is not possible with centralized PCR.

Future work should include improvement in three specific areas: (i) Sample collection and preparation, for example, using paper-based purification as well as DNA extraction with cell lysis;^59^ (ii) Reduction of incubation times for the assay by shortening the time of the SHA assay and by fully integrating the RPA in the paper; and (iii) Integration with open-source electronics to enable electrochemical readout with simple potentiostats^60^ and to automate the liquid handling steps, e.g., using electrical valves.^61,62^

## Supporting Information Available

Specification of threads used in this work, cyclic voltammograms of unmodified gold threads as working electrodes at various scan rates in tube- and paper-based systems, cyclic voltammograms of capture probe-modified gold threads as working electrodes at various scan rates in tube- and paper-based detection systems, sandwich hybridization assay (SHA) in tube- and paper-based detection systems with different volumes of the TMB + H_2_O_2_ enzymatic substrate, and Randles–Sevcik plots of capture probe-modified gold threads in the tube and paper-based systems (PDF)
