# Supplementary material for: Electroanalytical Paper-Based Nucleic Acid Amplification Biosensors with Integrated Thread Electrodes
Source: Anal Chem. 2021 Oct 14;93(42):14187–95. doi: 10.1021/acs.analchem.1c02900 (PMC8552215; doi:10.1021/acs.analchem.1c02900)
Supplement: Supplementary file 1 — ac1c02900_si_001.pdf [file ac1c02900_si_001.pdf]

Supporting Information

Electroanalytical Paper-based Nucleic Acid Amplification Biosensors with Integrated Thread Electrodes

Shirin Khaliliazar[1‡], Anna Toldrà[1‡], Georgios Chondrogiannis[1], Mahiar Max Hamedi[1*]

[1] School of Engineering Sciences in Chemistry, Biotechnology and Health, KTH Royal Institute of Technology, Teknikringen 56, Stockholm 10044, Sweden

(*) Author to whom correspondence should be addressed: mahiar@kth.se

(‡) Authors contributed equally to this work.

Table of Content

Table S1……………………………………………………………...……………..….2

Figure S1…..……………………………………………………………………………..3

Figure S2 ……………………………………………………………………………...4

Figure S3.. ……………………………………………………………………….....5

Figure S4.. ……………………………………………………………………..…...6

Figure S5.. ………………………………………………………………….……...7

Table S1. Threads used in this study and their characteristics.

Yarn type	Yarn Number	Filament. No	Diameter (µm)	Supplier
Gold Plasma coated yarn<br>Polyester FDY, high bright<br>(1.1 mg/m Au)	dtex 125/f36/2	72	246 ± 20	SWICOFIL AG
Silver Plasma coated yarn<br>Polyester FDY, high bright<br>(3.9 mg/m Ag)	dtex 125/f36/2	72	185 ± 19	SWICOFIL AG

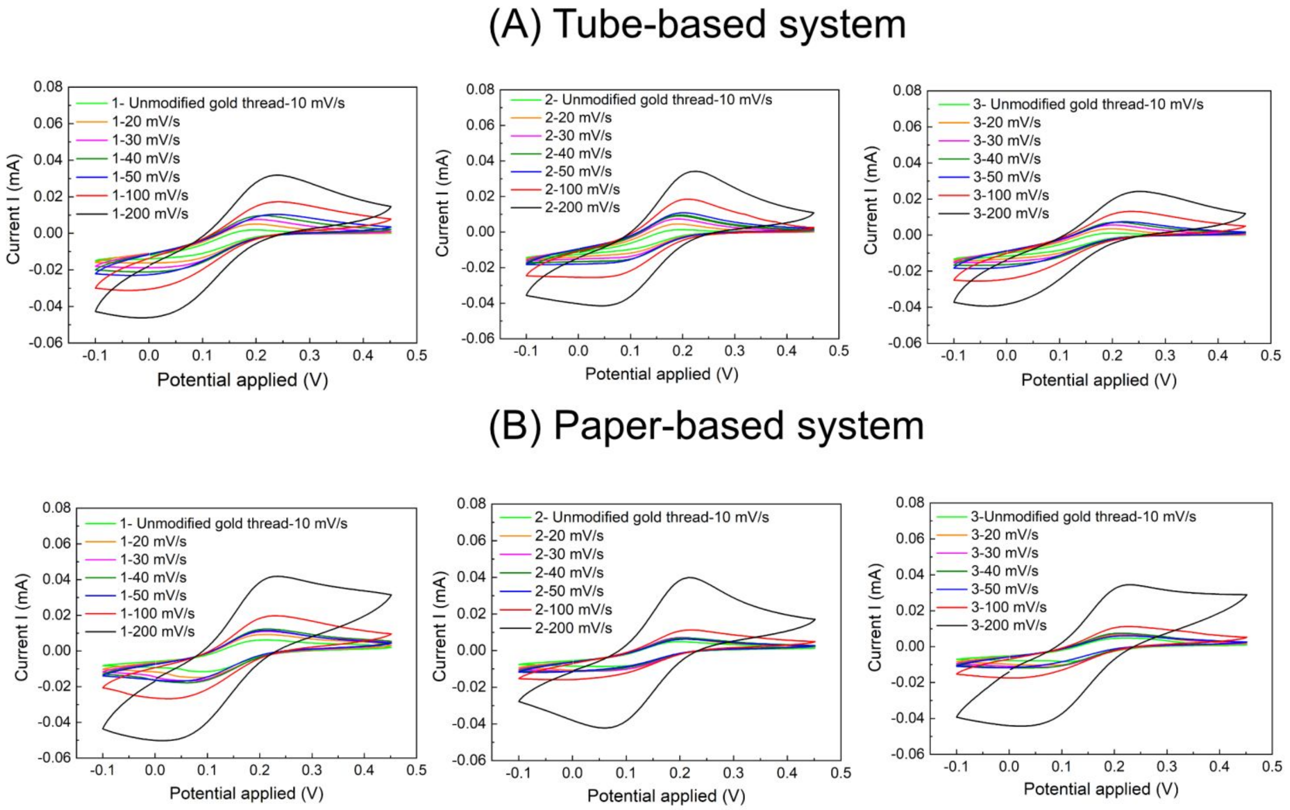

Figure S1. Cyclic voltammograms of unmodified gold threads (n=3) as working electrodes at various scan rates (10 mV/s - 200 mV/s) in presence of 5 mM of potassium ferricyanide solution using silver threads and gold threads as pseudo-reference and counter electrodes, respectively, for **(A)** Tube-based system; and **(B)** Paper-based system.

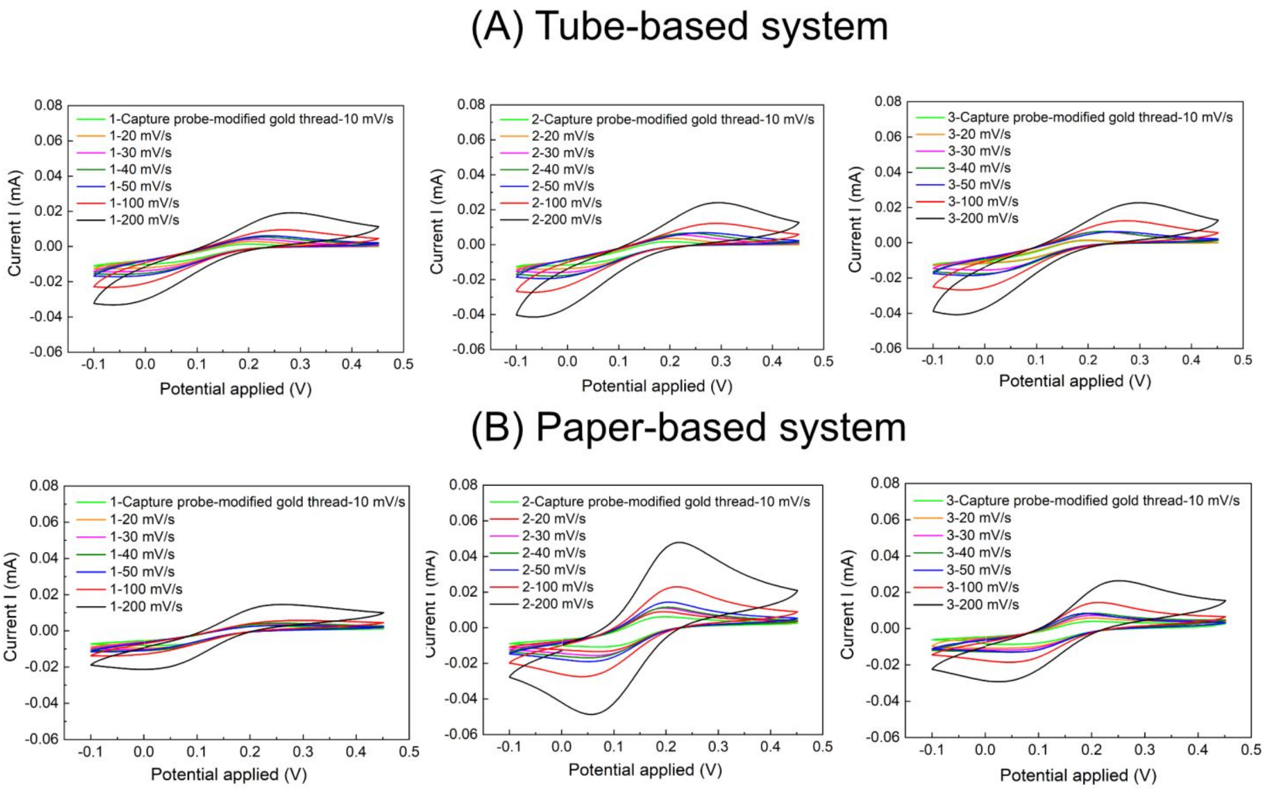

Figure S2. Cyclic voltammograms of capture probe-modified gold threads (n=3) as working electrodes at various scan rates (10 mV/s - 200 mV/s) in presence of 5 mM of potassium ferricyanide solution using silver threads and gold threads as pseudo-reference and counter electrodes, respectively, for: **(A)** Tube-based detection system; and **(B)** Paper-based system.

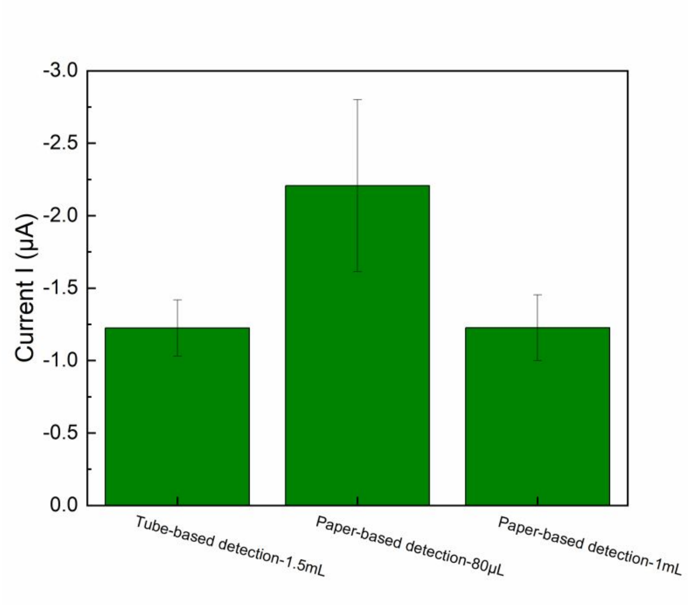

Figure S3. Sandwich hybridization assay (SHA) followed by tube and paper-based detection (n=3) with different volumes of TMB+H_2O_2 enzymatic substrate. Data are presented as mean ± standard deviation (SD).

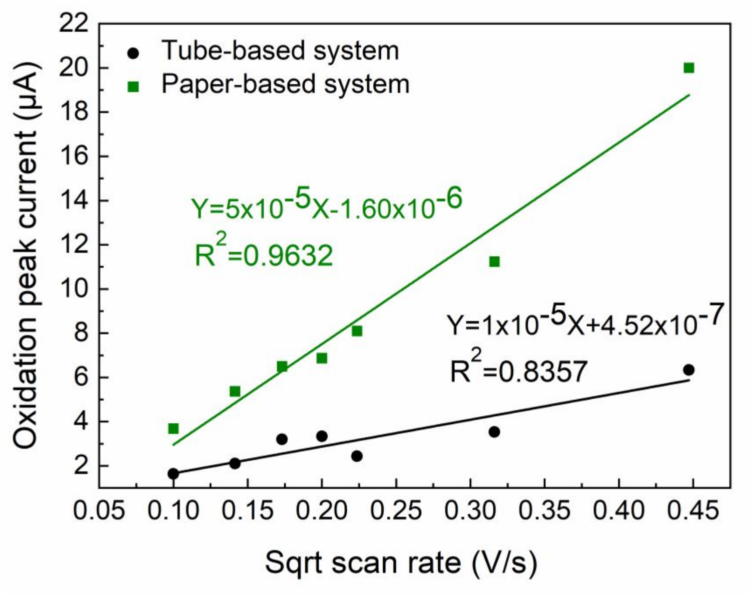

Figure S4. Randles-Sevcik plots of capture probe-modified gold threads (n=3) in the tube and paper-based systems. The electrochemical reaction in the paper-based system at the surface of the capture probe-modified gold threads can be characterized by a faster electron transfer (R^2 = 0.9632) than in the tube system (R^2 = 0.8357).

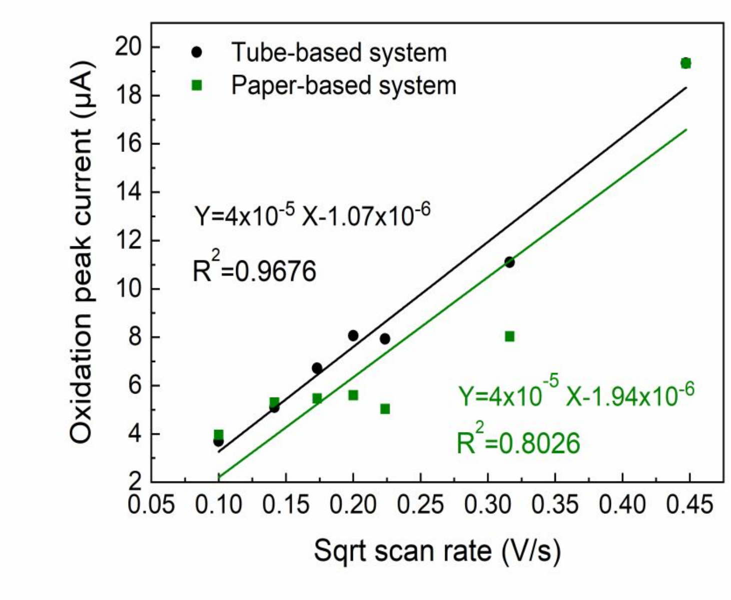

Figure S5. Randles-Sevcik plots of unmodified gold threads (n=3) in the tube and paper-based systems. The unmodified gold threads in the paper and tube-based showed contrariwise electrochemical behavior compared to their modified counterparts (see **Figure S4)**. The electrochemical reaction in the paper-based system at the surface of the capture probe-modified gold threads can be characterized by a slower electron transfer (R^2 = 0.8026) than in the tube system (R^2 = 0.9676).
